# Wolves lead and dogs follow, but they both cooperate with humans

**DOI:** 10.1038/s41598-019-40468-y

**Published:** 2019-03-07

**Authors:** Friederike Range, Sarah Marshall-Pescini, Corinna Kratz, Zsófia Virányi

**Affiliations:** 1 Wolf Science Center, Domestication Lab, Konrad Lorenz Institute of Ethology, University of Veterinary Medicine, Vienna, Savoyenstraße 1a, A-1160 Vienna, Austria; 2 Comparative Cognition, Messerli Research Institute, University of Veterinary Medicine, Vienna, Medical University of Vienna, University of Vienna, Veterinärplatz 1, 1210 Vienna, Austria

## Abstract

Due to their convergent evolution, dogs have been suggested as a good model for the evolution of human social skills, such as tolerance and cooperativeness. However, recent studies have revealed that wolves (dogs’ closest undomesticated relatives) are more tolerant and cooperative with conspecifics than dogs. It is still possible, though, that selection during domestication enhanced cooperative inclinations specifically towards humans, predicting better cooperation with humans in dogs than in wolves. We tested this hypothesis by comparing similarly human-raised wolves and dogs when cooperating with a familiar human partner in a string-pulling task. Both dogs and wolves were highly successful with the human partner, highlighting that dog-human cooperation could have evolved based on wolves’ social skills. However, wolves and dogs differed in how they cooperated with their human partners with wolves being more likely to initiate movement leading the interaction with humans, whereas dogs were more likely to wait for the human to initiate action and then follow. Accordingly, we propose that during the course of domestication, after an initial reduction in fear of humans, dogs were selected for increased submissive inclinations (Deferential Behaviour Hypothesis) in order to minimize conflicts over resources, to ensure safe co-habitation and co-working in a way that humans lead and dogs follow.

## Introduction

Human social life heavily depends on cooperation and the frequency and complexity with which humans cooperate with each other are exceptional, if not unique. To understand the evolution of our exceptional skills, researchers have suggested dogs (*Canis familiaris*) as a good model of human cooperation based on humans and dogs having been exposed to similar environmental pressures, thereby potentially representing an example of convergent evolution^1–3^. As such, it has been proposed that during the domestication process, dogs have acquired specific predispositions for cooperative interactions due to reduced aggression and increased tolerance^4,5^. This would predict more successful cooperation in dogs than in wolves. However, wolves are a highly cooperative species: cooperating during raising of the young, hunting and territory defence^6^. Accordingly, we have suggested that dogs did not develop novel traits during domestication but rather that the intraspecific cooperation abilities of their common ancestor (wolves) provided the foundation for the evolution of dog-human cooperation (Canine Cooperation Hypothesis)^7^. In line with the idea, our previous results on cooperative interactions between conspecifics using the loose-string paradigm revealed that wolves can be highly successful in simultaneously pulling the two ends of a rope to move a tray close enough to get access to the food positioned on top^8^. Interestingly though, in this task, our dogs performed rather poorly, which could be due to a reduction either in their cognitive understanding of different components of the task or in their tolerance towards conspecifics preventing dogs from working simultaneously at the apparatus^9^. This latter suggestion is further supported by the fact that highly trained pet dogs do cooperate with each other at least in their owner’s presence that may mediate tolerance between the dogs^10^.

These previous results raise a few additional questions regarding wolves’ and dogs’ cooperative abilities.

Despite dogs show limited cooperation with conspecifics in comparison to wolves, it is still possible that domestication has led to changes that enhance cooperation specifically with humans. Although the mutualistic aspect of dog-human cooperation has yet to be shown, the multiple roles in which dogs assist humans are ubiquitous and invited plenty of research (see for example^11^). To explain the success of dogs in such roles, a number of domestication hypotheses have been proposed suggesting that dogs have evolved higher attentiveness towards humans^12,13^, lower aggression^3,14^, increased inhibitory control^15^, higher sociability^16,17^ or higher social competence^18^ when interacting with them. These hypotheses predict that dogs will outperform wolves when cooperating with humans whereas based on the Canine Cooperation Hypothesis we expect that, if early and intensive socialization with humans is given, wolves can cooperate *with humans* as well as dogs.

In the present experiment, we tested wolves and dogs at the Wolf Science Center, Austria, where animals are similarly socialized, from a very early age, with humans. To test the different hypotheses, we compared their performance in the cooperative loose-string paradigm with familiar human partners. Successful cooperation was defined as the animal coordinating its actions with the human partner so that they pulled simultaneously on the two ends of the rope thereby moving the platform forward allowing them to access the out-of-reach food. If only one partner pulled, the string would come loose and the trial was coded as a failure. To investigate whether and how the animals adjusted to the human cooperation partner, we ran two experimental conditions. In the first, *spontaneous condition*, we released the human and animal partners in a way that in half of the trials the animal and in the other half the human would arrive slightly before the partner and thus could choose which end of the rope to pull on. While in the first situation, the animal had ‘merely’ to go to its preferred side and wait for the human partner to arrive and then pull simultaneously, in the latter situation, to be successful, the animal had to adjust its behaviour to that of the human and pull on the non-preferred side. If they did instead try to take the position of the human partner by stealing the rope, the human partner refused further cooperation and the trial failed. In the *dual tray condition*, we used two loose-string trays placed a considerable distance apart (10 m), to investigate how the animals would coordinate their behaviours with the human partner when needing to decide which tray to tackle first and how to move from the first to the second apparatus e.g. whether they would initiate movement to the second apparatus or follow the human by waiting until the human left the first apparatus and then follow the human within 1 body length. Only if *both* apparatuses were solved, the trial was counted as being successful. In both experiments, the human partner was instructed not to communicate with the animal and once touching the rope, either to pull in unison with the animal or pull no matter what after 3 seconds.

## Results

**Figure 1:**
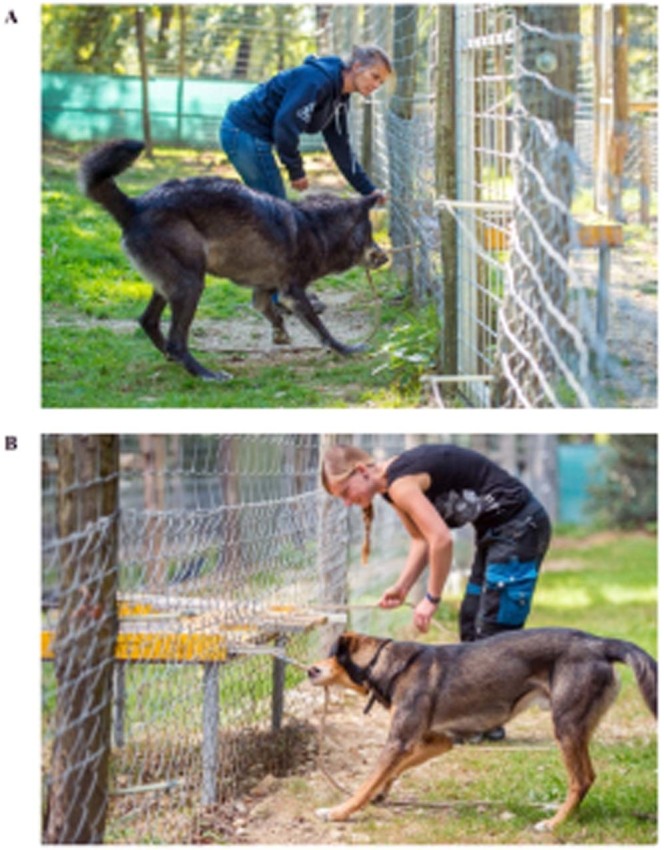
Illustration of a wolf (**A**) and a dog (**B**) working with a human cooperation partner (photographs by Rooobert Bayer).

15 grey wolves (11 males, 4 females, age: 2 to 8 years) and 12 mixed-breed dogs (7 males, 5 females, age: 2 to 7 years) housed at the Wolf Science Center (WSC) in Ernstbrunn, Austria, participated in loose-string experiments together with a human partner (Fig. 1), who, by working as a ‘trainer and keeper’ at the WSC, had extensive daily contact with the animals in a variety of settings (leash walking, tests, animal care, hand-raising etc.) thereby establishing a close affiliative bond with them. Each animal was tested with the trainer it had the best relationship with. The trainer was kept constant across all sessions, but trainers varied between animals.

All animal-human dyads were first exposed to 6 sessions of 6 trials each of the ‘classic’ version of the loose-string paradigm^19–24^, where a single tray was presented (*spontaneous condition*). At the start of each test trial, the animal subject (either wolf or dog) and the human cooperation partner were positioned in the shifting system facing the test tray at a distance of 40 m. In 50% of the trials, we released the animal when the human partner had reached the middle of the enclosure so that the animal reached the tray approximately 3 seconds before the human cooperation partner. In these trials, the animal could choose the side it wanted to pull the rope on, and then wait for the human partner to arrive and pull the other rope. In the other 50% of the trials, the animal was released when the human cooperation partner was only 3 meters away from the tray, so that the human cooperation partner arrived approx. 3 seconds before the animal at the tray and could choose which rope to pull on. The human cooperation partner was not allowed to talk, look or gesture to the animal in any way during a trial and followed a strict protocol (see SI Materials and Methods for details on the procedures and protocol) so that the animal had to coordinate with the human partner and not vice versa.

Since all subjects except 3 wolves had previously participated in the string-pulling paradigm with conspecific partners and thus had different experiences^8^, in the analyses, we included two variables that captured the more salient aspects of their previous experience with the task: (a) training: i.e. whether animals had received individual training to hold both ends of the rope in their mouth and pull the tray forward before being tested with a conspecific partner and (b) previous success: the average percentage of trays successfully solved across testing in all conditions with all previous conspecific partners (i.e. if an individual was tested with 3 different partners in the one-tray condition, with 2 of these partners in the two-apparatus condition, and once in the delay condition where the partner was released 10 seconds later, the individual’s score was calculated as the mean percentage of success across these 6 partners/conditions)(see^8^)(see ST9 for a list of the animals with their respective experiences).

We found that when tested with a human partner in the *spontaneous condition*, wolves succeeded above chance level: on average in 61.5% of trials (range: 25–91%, binomial test: p < 0.001; see ST 11 for the means and SDs of the coded behaviours). Dogs on the other hand performed at chance level i.e. on average they succeeded in 49% of trials; range: 0–88%, (binomial test p = 0.32). Only one animal never succeeded in solving the task with the human partner (Imara, a dog). A Generalized Linear Mixed Model (GLMM- proportional data- binomial distribution) with number of successes (defined as getting access to the food by pulling the rope simultaneously with the partner) in each session as the dependent variable, species, session, previous success and training, as the explanatory factors and animal ID as the random factor was run, followed by a model comparison approach based on AICc (MuMin package in R)(see Supplementary Information for details of the statistical analyses). The best model showed a strong effect of session, with success increasing across sessions (for model comparisons see Supplementary Table 1(ST1). Furthermore, having successfully cooperated with conspecifics in the previous experiment (‘previous success’) also weighed on the likelihood of success, but to a lesser extent (Table 1). Species and prior training on the tray had very little impact on the animals’ rate of success.

**Table 1:** Estimated effect size, adjusted standard error (SE), z-value and relative variable importance (RVI) estimated by a generalized linear mixed model to determine the effects of session (each session compared to session 1), training, previous success and species on the likelihood of success in the spontaneous condition.

	Effect size	Adj. SE	z value	P	RVI
Session 2	0.41	0.261	1.558	0.119	0.86
Session 3	0.61	0.263	2.333	**0.019**	0.86
Session 4	0.51	0.262	1.946	**0.051**	0.86
Session 5	0.61	0.263	2.333	**0.019**	0.86
Session 6	0.97	0.267	3.619	**0.0002**	0.86
Prev. Success	0.02	0.009	1.99	**0.047**	0.68
Species	0.55	0.63	0.88	0.379	0.34
Training	0.19	0.55	0.34	0.733	0.26

Animals were significantly more successful in trials in which they arrived at the tray before the human did (wolves: mean animal first 88% vs. human first 50%; dogs: mean animal first 70% vs. human first 40%)(Wilcoxon: V = 263, p-value = 0.0002) highlighting the difficulty for the animals to give up their own preferences and adjust to the human. Furthermore, we ran a number of GLMMs to evaluate whether wolves and dogs differed in their behaviors with the human partner during the trial namely (a) the frequency of gazing at the human partner (b) and the likelihood of stealing the rope from the human partner, which usually happened when the human was at the tray first and chose the animals’ preferred rope-side. Session and previous success were included as explanatory factors in all models, as well as animal ID as random factor. Frequency of gazing was normalized by the time spent within one body length of the tray (using the offset function), since the behavior was recorded during the time the animals were in front of the tray (and hence within view of the camera).

The average frequency of looking to the human partner per trial was 0.3 times (range: 0–8) for wolves and 0.7 times (range 0–10) for dogs. An interaction between who arrived first (human vs. animal) at the tray and species emerged on the frequency of looking at the human partner (GLMM: Chisq = 4.989, df = 1, p = 0.026). In trials, where the human arrived first, there was no effect of species, but an effect of previous success and session (Table 2, model comparisons: ST2) with animals that had less experience looking more at the human, and an overall increase in looking at the human partner across sessions.

**Table 2:** Estimated effect size, adjusted standard error (SE), z-value and relative variable importance (RVI) estimated by a generalized linear mixed model to determine the effects of session, species and previous success on gazing at the human partner in trials in which the *human* arrived first at the tray.

	Effect Size	Adj. SE	z value	P	RVI
Prev. success	−2.946469	0.345245	8.534	**<0.001**	0.94
Session	0.110677	0.039715	2.787	**0.005**	0.86
Species	−0.677061	0.487824	1.388	0.165	0.52

In trials, in which the animal arrived first at the tray there was no effect of previous success or session, but wolves looked at the partner significantly less frequently than dogs (mean looking at partner: wolf: 0.54, range 0–8; dog: 1.2, range 0–10)(Table 3, model comparisons: ST3).

**Table 3:** Estimated effect size, adjusted standard error (SE), z-value and relative variable importance (RVI) estimated by a generalized linear mixed model to determine the effects of session, species and previous success on gazing at the human partner in trials in which the *animal* arrived first at the tray.

	Effect Size	Adj. SE	z value	P	RVI
Prev. success	−0.006826	0.005818	1.173	0.241	0.38
Session	−0.032406	0.036113	0.897	0.369	0.35
Species	−0.598522	0.235041	2.546	**0.011**	0.54

Since, if animals arrived at the tray first, the person was instructed to operate the opposite (free) rope, no opportunity for stealing the rope from the human arose in these trials. So we limited our analyses of rope-stealing to trials in which the human arrived first. We found that 14 out of 15 wolves stole the rope in at least one trial (mean: 6.9; range 0–18), while only two out of 12 dogs stole the rope (one dog in two and one in five trials). Overall, wolves stole the rope in 19% of trials and thus were more likely to steal the rope from the human than dogs; however, this behavior decreased across sessions and moreover, it was not affected by previous success (Table 4, model comparisons: ST4).

**Table 4:** Estimated effect size, adjusted standard error (SE), z-value and relative variable importance (RVI) estimated by a generalized linear mixed model to determine the effects of session, species and previous success on likelihood of the animals stealing the rope from the *human* partner.

	Effect Size	Adj. SE	z value	P	RVI
Previous success	−0.01030	0.01005	1.025	0.305	0.38
Session	−0.18085	0.06159	2.936	**0.003**	0.97
Species	2.98574	0.78271	3.815	**<0.001**	1

Nine wolves (7 males, 2 females, age: 2 to 8 years) and 7 dogs (4 males, 3 females, age: 2 to 7 years) solved the single tray with the human partner on at least 4/6 trials in 2 consecutive sessions and thus also participated in the *dual tray condition* with the exception of two wolves that despite reaching criterion were not tested further due to them being uncomfortable with unfamiliar people, which would have required trainers to run the entire testing. In this condition, the human was released first and approached the trays from the mid-line, only starting to directly approach the *a priori* designated tray, when reaching the second half of the enclosure. The animal was always released when the human had reached the middle of the enclosure and thus, while it had to adjust to the human in regards to which tray to solve first, given the greater speed of the animals in moving, it could arrive first and choose which rope-end to pull. If the animal was the first to reach the designated tray, the human partner took the rope-end not chosen by the animal. If the human cooperation partner reached the first tray before the animal, she chose the side of the rope ***not*** preferred by the animal based on side preferences observed in the previous condition. Since in this condition we were primarily interested in whether the animals would coordinate their behaviour with the human partner and approach the same tray, we opted not to force them to also use their non-preferred side to pull the rope.

If the human cooperation partner and the animal solved the first tray successfully or if the rope was pulled out or stolen by one of the partners, the human cooperation partner either (1) followed the animal, if it moved towards the second tray or (2) waited for 5 seconds before approaching the second tray. Apart from these differences, the same protocol was followed as for the first experimental condition (see details of the experimental procedure in the Suppl. Material and ST 12 for the means and SDs of the coded behaviours).

Wolves successfully solved both trays in 76% (range 44–94%) and dogs in 67% (range 55–77%) of trials (both binomial tests: p < 0.001). A Generalized Linear Mixed Model (GLMM) with the number of successes (i.e. solving both trays in a trial) in each session as the dependent variable, species and session as the explanatory factors and animal ID as the random factor followed by a model comparison approach based on AICc (package MuMin in R) revealed an effect of session but not species on the likelihood of success in the *dual tray condition* (see Table 5, model comparison: ST5), with animals becoming more successful across sessions.

**Table 5:** Estimated effect size, adjusted standard error (SE), z-value and relative variable importance (RVI) estimated by a generalized linear mixed model to determine the effects of session and species on success in the dual tray condition.

	Effect size	Adj. SE	z value	P	RVI
Session	0.23548	0.096	2.463	**0.014**	0.88
Species	0.13353	0.154	0.865	0.387	0.29

Since the human partner was released first and hence chose which tray to go to, following the human was a crucial aspect of the task. Following to tray 1 was defined as moving with the partner (within 1 body length), however the animals could still arrive and solve tray 1 successfully and not follow the person (so for example by visiting tray 2 first or lagging behind and then quickly joining the human on tray 1) or they could follow and then not be successful (by for example not pulling the rope with the partner). Overall, tray 1 was successfully solved on 190 of a total 252 trials and of these 190 trials in which animals successfully solved tray 1 they followed the partner in 140 trials. They followed but failed in 10 trials. We ran a GLMM with ‘following the human to tray 1’ as the dependent variable. We found no effect of species (wolves followed the human partner in 62% and dogs in 60% of trials), but an effect of session (with an increase across sessions) on the likelihood of following the human partner to the first tray (Table 6, model comparisons: ST6).

**Table 6:** Estimated effect size, adjusted standard error (SE), z-value and relative variable importance (RVI) estimated by a generalized linear mixed model to determine the effects of session and species on the likelihood of following the human partner to the first tray in the dual tray condition.

	Effect Size	Adj. SE	z value	P	RVI
Session	0.33	0.162	2.041	**0.041**	0.75
Species	0.068	0.27	0.250	0.802	0.27

Having (successfully or unsuccessfully) completed tray one, two scenarios could emerge: the animal could wait (for 5 seconds) for the human partner to move towards the other tray or take the lead itself. We therefore ran a second analysis with the likelihood of ‘leading to tray 2’ as the dependent variable. Finally, if the human (rather than the animal) took the lead, the subject could choose to follow (defined as staying within 1 body length of the human cooperation partner) or not (for example lingering longer at tray 1 and/or sniffing around the enclosure). Neither leading nor following the partner to tray 2 equated with success, since once the animals reached the tray, they still needed to coordinate their pulling actions with the partner. At the same time, the dyad could still be successful if the animal did something else while the human moved to the second apparatus, but then ran over to pull the second end of the rope just in time.

**Figure 2:**
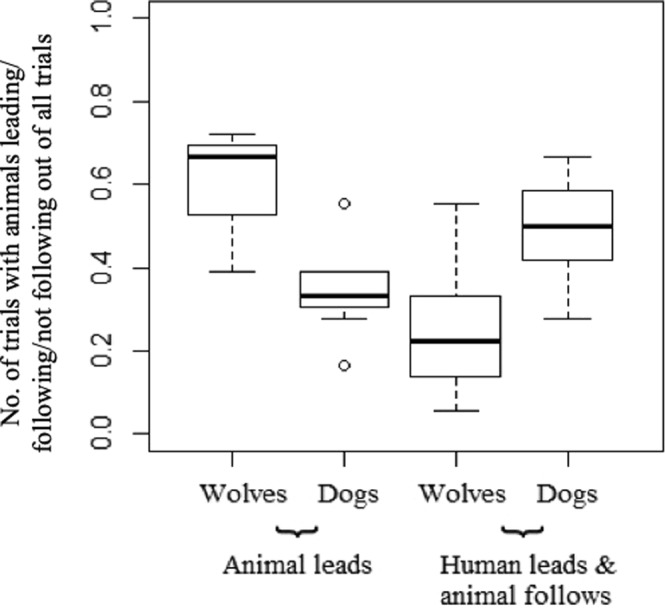
Visualizing the differences between wolves and dogs in leading from the first to the second apparatus (animal leads) and whether or not the wolves and dogs would follow a human leading from the first to the second apparatus. *Represent statistical differences.

Overall, the animals solved at least tray 2 in 220 of 252 trials. Of these 220 trials, animals led in 119 and followed in 67 trials. Of all the trials they led (120) they failed only one. They followed the human on 78 trials in total and failed in 11 of these. We ran a final GLMM on a subset of the data considering only trials in which the human led the way from the first to the second tray and including ‘following the human to tray 2′ as the dependent variable. Wolves were more likely than dogs to take the lead in moving from the first to the second tray (wolves took the lead in 60% and dogs in 35% of trials) (Fig. 2). While taking on the leading role increased across sessions (Table 7, model comparisons: S79), the difference between wolves and dogs remained significant also in the last session (see Supplementary Results). Moreover, previous experience of the wolves with the two-tray condition with conspecifics did not influence the likelihood that a wolf took over the leading role of the human (see Supplementary Results).

**Table 7:** Estimated effect size, adjusted standard error (SE), z-value and relative variable importance (RVI) estimated by a generalized linear mixed model to determine the effects of session and species on the likelihood of the animal leading from the first to the second tray in the dual tray condition.

	Effect Size	Adj. SE	z value	P	RVI
Session	1.109	0.326	3.404	**<0.001**	0.98
Species	0.508	0.168	3.015	**0.003**	0.97

Finally, in trials in which the human led from the first to the second tray (N = 50 for wolves and N = 82 for dogs), we found that the dogs were more likely to follow the human partner (doing so in 70% of trials) than wolves (which followed in 42% of trials). Again, while the likelihood of following increased across sessions (Table 8, model comparison ST8), the difference between wolves and dogs also remained significant in the final session and was independent of previous success in the wolves (see Supplementary Analyses).

**Table 8:** Estimated effect size, adjusted standard error (SE), z-value and relative variable importance (RVI) estimated by a generalized linear mixed model to determine the effects of session and species on the likelihood of the animal following the human from the first to the second tray in human-lead trials.

	Effect Size	Adj. SE	z value	P	RVI
Species	−1.2376	0.6209	1.993	**0.046**	0.88
Session	0.6367	0.2684	2.372	**0.018**	0.67

## Discussion

Overall, the data presented here reveal that when socialized with humans and kept under similar conditions, despite dogs showing deficits when cooperating with conspecifics compared to wolves, dogs and wolves do not differ in their ability to successfully cooperate with a familiar human partner. However, interesting differences between wolves and dogs emerge when the details of the cooperative interactions are analyzed, showing that while wolves are more inclined to initiate behavior and take the lead, dogs are more likely to wait for the human partner to initiate going to the second tray and then follow.

To date, the most prominent domestication hypotheses have suggested that during domestication, dogs have been selected for certain skills that allow them to communicate and cooperate with human partners^3,12,13^, a view that has been widely accepted without the necessary scrutiny and evaluation of the predictions following on from such hypotheses. Only few studies have compared wolves and dogs that have had the same experiences with human partners throughout their lives, and most of these studies have tested animals younger than 4–6 months^15,25,26^. At this age canines are still considered juveniles or even puppies with their social skills and cognitive capacities not fully developed^27,28^. A few other studies have tested highly socialized adult wolves and compared them with shelter or pet dogs^13,16,17,29,30^. Overall, most of these studies have found more human-directed behavior and/or better communicative skills in dogs than wolves (but see^31^: although differential raising and experience with humans make results difficult to interpret).

In contrast, the wolves and dogs at the Wolf Science Center are highly socialized with humans and conspecific partners and importantly, have had the same experiences throughout their lives, allowing us to make a fair comparison when trying to understand differences in how they interact with both conspecific and human partners. Not surprisingly given the highly social cooperative environment of wolves^6^, our wolves’ success does not differ from dogs when presented with a task that requires cooperation with a familiar human partner. Wolves and dogs were successful on average in 61% and 49% of the trials in the spontaneous condition despite having no previous experience of this task with a human partner. This is in line with several recent studies that compared the wolves and the dogs at the Wolf Science Center in interactions with human partners. In regard to interactions with humans, adult wolves and dogs can use the information provided by a human partner to find a hidden food reward to a similar extent (gaze following and pointing in a two choice task^32^, local enhancement^33^). Moreover, they can differentiate between cooperative and uncooperative human partners, showing (i.e. gaze alternating) a hidden food reward predominately to the cooperative partner^34^. These studies suggest that wolves, as dogs, can accept humans as a social partner when highly socialized.

It is important to note that the success of wolves in the current cooperative string-pulling task cannot be explained by their former, overall more successful experiences with conspecifics^8^. In fact, to control for the differential experience of dogs and wolves with the task, we included previous success in the statistical analyses and no species effect emerged. All animals improved over sessions showing a clear learning effect and regardless of species, previous success in the string-pulling task with a conspecific partner, positively influenced their performance. Moreover, the three wolves that had no prior training nor previous experience with the task were actually quite successful (Etu 36%, Maikan 53%, Tekoa 92%) with Tekoa outperforming all other animals except one other wolf, that performed as well as he did (Chitto). Interestingly, these three naïve wolves were also more successful than wolf-wolf dyads that were completely task naïve (Kaspar-Shima 55.5%, Chitto-Tala 8%, Gero-Amarok 0%, Nanuk-Una 6%, Wamblee-Yukon 5%^8^), suggesting that cooperating with humans rather than conspecifics might be easier for wolves as well as for dogs at least in this specific setting.

The finding that less task-experienced animals gazed more at the human partner than those with more experience in the trials of the spontaneous condition, where the human arrived first, may suggest that looking at the human could help to better coordinate with the partner. Alternatively, looking at the human may be interpreted as a help-seeking behavior, as proposed by former studies testing dogs and wolves in an unsolvable task^12^. In the current study, we found that dogs looked at their human partner significantly more often than the wolves only in trials, where the animal arrived first to the tray. This species difference could be due to wolves being more manipulative/explorative than dogs and thus, engaging in interacting with the apparatus rather than looking at the human^29,35–39^. This could also be due to dogs being more dependent on humans than wolves^12,40^, but looking back behavior in adult animals does not seem to indicate dependency on humans or help seeking behavior^41^, and has been shown to be equally frequent in adult wolves and dogs in another context^34^. Independent of the reason, the dogs’ looking behavior might have enabled them to coordinate properly, whereas for the wolves, fewer looks were sufficient.

A much stronger difference that we found between wolves and dogs in the spontaneous condition is the rope stealing behavior that was mainly exhibited by the wolves. Important to note here is that while the dogs did not steal the rope, they were also not more successful than the wolves if the human arrived first, suggesting that they did not display this behavior because they were more flexible than the wolves in changing sides and pulling the free rope. They just did not try to take the rope away from the human partner. This difference nicely fits in with the behavior of the animals shown in the dual tray condition. In the *dual tray* condition, while we still did not find differences in the wolves’ and dogs’ capacity to coordinate with a human to solve both trays in a trial, wolves and dogs seemed to differ in how they reached this goal. While the wolves initiated movement from the first to the second tray and were less likely to follow the human partner when she moved first, dogs were more likely to wait for the human partner to initiate going to the second tray and then followed, suggesting that the wolves adopted a leading, while the dogs adopted a following role during this cooperative event. This assertiveness of wolves is also evident in most of them trying to steal the rope from the human partner in the *spontaneous condition* to pull on their preferred side. Interestingly, they decreased this behavior over trials probably due to learning that if they were being uncooperative in that aspect, they were unsuccessful due to the human partner withdrawing her help, resulting in not getting access to the food.

Placing it into a broader context, the dogs’ behaviors towards their human cooperating partners, when compared to that of wolves, appear to be more ‘deferential’ (gazing at the human, not stealing the rope, waiting for the human to initiate the action and then following her to the second tray) and they appear consistent with the ‘conflict-avoidance’ strategy dogs show when interacting with conspecifics. During pack feeding, subordinate dogs often do not even try to come close to the food source, when a more dominant animal is present and dominant dogs show more agonistic behaviors than subdominant pack members^42,43^. In contrast, dominant wolves in these situations tolerate subdominant pack members and the latter more often show agonistic behaviors and attempts at obtaining food than dogs. Interestingly, also in non-feeding contexts wolves’ and dogs’ conflict-related behaviors differ. While wolves generally have more conflicts, dogs engage significantly more often in high intensity conflicts involving physical contact^44^, leading to potentially higher risks of injury. Not surprisingly given these observations, wolves reconcile with their former opponents, while dogs instead keep a greater distance from one another after conflicts occur^44^. These results on conspecific interactions suggest that wolves and dogs use two different conflict management strategies with wolves engaging in conflicts but then using affiliative behaviors to resolve them in a proactive manner, while subdominant dogs appear to try to avoid conflicts by keeping distance from resources ‘taken’ by the dominant animal, and also using distance maintenance as a post-conflict strategy, which hints towards differences in the social ecology of wolves and dogs (see^6^ for a discussion). These results suggest that the failure of the dogs in the string-pulling task with conspecifics was likely due to the low tolerance of dominant animals and the tendency of subdominant dogs to avoid potential conflicts around a resource, making it difficult for the animals to simultaneously pull the two ropes connected to the food tray (see also^8,9^). This can also explains why the dogs were immediately more successful when cooperating with human partner (dogs, even with extensive training (see^45^), were successful on only 20% of trials with other pack members but they increased to an average of 49% successful trials when paired with a human partner in the spontaneous condition). Their higher success with the human partner is probably a result of the positive, non-competitive experiences these dogs have had with humans throughout their lifetime resulting in them not perceiving a risk of conflict over the food on the table, and hence not needing to adopt distance maintenance as a conflict preventive strategy. This in turns facilitates the manifestation of a more cooperative strategy, based on closely monitoring (looking at) and following the human partner, and adapting to their behaviour. Such a human influence might have contributed to the successful cooperation a former study reported between highly trained pet dogs living in the same household^10^. The presence of the owner during the test as well as life-long socialization by humans mitigating tolerant behavior might have increased the chance of these dogs to succeed.

Alternatively, one may suggest that it is the wolves’ and dogs’ cognitive capacities what differ. If wolves have a better understanding of the string-pulling task than dogs for example due to better casual understanding^32^, they may more easily overcome other obstacles (such as low tolerance) to solve the task. Dogs on the other hand may need to rely more on a ‘predictable’ partner (i.e. someone who will wait 3 seconds and start pulling) to accomplish the task. This interpretation would be in line also with the results that wolves lead and dogs follow. The fact that also the younger wolves showed increased gazing at the human partner, but also the highest success, suggest that cooperation with such ‘predictable’ human partners is in fact easier than cooperating with conspecifics. However, a follow up study revealed that wolves and dogs perform similarly well when they need to asses whether solving the apparatus needs a partner or not and recruit a human partner with similar success (Range *et al*., submitted), suggesting that dogs had a similar understanding of the task as the wolves.

Other behavioral changes proposed by the various domestication hypotheses (better inhibitory control, reduced aggression and heightened sociability) to have enabled dog-human cooperation, cannot explain the current results. If wolves had worse inhibitory control than dogs (‘synergistic hypothesis’^15^), we would expect them to be less successful than dogs in this task, since they would have found it harder to inhibit rope pulling, which was clearly not the case. In fact, two other studies have shown no clear differences in inhibitory control abilities between adult wolves and dogs^46,47^. Moreover, none of our wolves engaged in aggression towards the human partner (‘emotional reactivity hypothesis’^3,5^). If at all, the previous results with the conspecific partners suggest that it is dogs that were more careful towards their partners possibly expecting aggressive interactions. Sociability *per se* also cannot explain our results, since again, if cooperation were enhanced by dogs increased closer proximity to the human partner compared to wolves, we would have seen a higher success rate in dogs than in wolves (‘sociability hypothesis’^16^).

Taken together, our results lend support to the idea that dogs’ abilities to cooperate with humans largely derive from wolves’ (intraspecific) cooperative skills (Canine Cooperation Hypothesis), since when intensely socialized, wolves, have all the necessary skills (attentiveness, tolerance, inhibition) to successfully cooperate not just with conspecifics, but also with a familiar human partner. Thus, while selection for reduced fear of humans was certainly an important step during domestication, resulting in less human-socialization time being necessary for dogs compared to wolves to obtain a ‘socialized’ animal, no specific cooperative skills needed to evolve in dogs to enable cooperation. Nevertheless, the Canine Cooperation Hypothesis cannot fully account for the differences in how wolves and dogs cooperate either with humans or with conspecifics. In fact, if tolerance over a resource is seen as an intrinsic part of cooperation, then dogs can be considered less cooperative than wolves. Moreover, the reduced rope-stealing, more following, less leading, more looking behaviors in dogs than in wolves in the current study also suggest that dogs did evolve a different, more compliant, style of coordinating with humans. Overall, the social interactions of dogs and wolves show consistent differences both in intra- and interspecific contexts indicating that dogs may be more likely to avoid conflicts and follow the human’s lead, thus cooperating in a deferential and adaptive manner rather than as equal partners. Accordingly, we propose that after an initial selection against fear during the domestication process, dogs were selected for increased deference (Deferential Behaviour Hypothesis) in order to minimize conflicts over resources, to ensure safe co-habitation and co-working in a way that humans lead and dogs follow. Future studies will need to address what kind of human-dog interactions were enabled by these changes and whether and under what conditions these interactions can be considered as a form of cooperation benefiting both parties.

## Materials and Methods

Details of the subjects, testing, training, coding of test and observations, as well as statistical analyses carried out are included in the SI Materials and Methods, Fig. S1, Tables S1, S2, Movies S1, S2 and Dataset S1. This study was discussed and approved by the institutional Ethics and Animal Welfare Committee at the University of Veterinary Medicine Vienna, in accordance with Good Scientific Practice guidelines and national legislation

(Protocol Number: ETK-01/04/97/2014 & ETK-09/09/2018).

All humans gave informed consent for participating in the experiments and the people visible in Fig. 1 and Videos 1 and 2 gave informed consent for the publication of these materials in an online open-access publication.

## Supplementary information

Range ef al. Supplementary Information

Movie S1

Movie S2

Complete Data Set
