# Supplementary material for: Wolves lead and dogs follow, but they both cooperate with humans
Source: Sci Rep. 2019 Mar 7;9:3796. doi: 10.1038/s41598-019-40468-y (PMC6405935; doi:10.1038/s41598-019-40468-y)
Supplement: Supplementary file 1 — Range ef al. Supplementary Information [file 41598_2019_40468_MOESM1_ESM.doc]

**Supplementary Information**

Wolves lead and dogs follow, but they both cooperate with humans.

**Friederike Range^1,2*+^, Sarah Marshall-Pescini^1,2*^, Corinna Kratz^1^, Zsófia Virányi^2,1^**

## Supplementary Results

Statistical Analyses

Since all the subjects had had different experiences with the string-pulling tray prior to being tested with the human, we controlled for this in our analyses by inserting two variables which captured the more salient aspects of their experience with the task: a) training: i.e. whether animals had received individual training to hold both ends of the rope in their mouth and pull the tray forward before being tested with a partner (see ^44^) and b) previous success: calculated as the number of trays solved over the total number of trays presented to the subject (in spontaneous, dual and delay conditions) with all previous conspecific partners ^8^.

***Spontaneous Condition***

A Generalized Linear Mixed Model (GLMM- proportional data- binomial distribution) with number of successes in each session as the dependent variable, species, session, previous success and training, as the explanatory factors and animal ID as the random factor was run. A Wilcoxon test was run to evaluate whether animals were more successful in trials in which they arrived first at the tray vs. when the human arrived first. Two further GLMMs were run to evaluate whether wolves and dogs differed in their behaviors with the human partner during the test. A GLMM with a Poisson distribution was run with the frequency of gazing at the human partner in each trial (normalized by the time spend in proximity to the apparatus- using the offset function), as the dependent variable and a GLMM with a binomial distribution was run with whether the animal did or did not steal the rope from the partner in a trials as the dependent variable. For both these models species, session, previous success and training, were entered as the explanatory factors and animal ID as the random factor.

***Dual tray condition***

Success was defined as the subject and partner solving both trays in a trial. A Generalized Linear Mixed Model (GLMM) was run with the number of successes in each session as the dependent variable, species and session as the explanatory factors and animal id as the random factor.

Three further GLMMs were run also to evaluate whether wolves and dogs differed in their cooperative behaviors with the human partner during the trials. Since the human partner was released first and hence chose which tray to go to, following/synchronizing movement with the human was a crucial aspect of the task. We therefore ran a model with whether or not in a trial the animal ‘followed the human to tray 1’ as the dependent variable. Having completed tray one, two scenarios could emerge; the animal could wait for the human partner to move towards the other tray, or take the lead itself. We therefore ran a second analysis with the occurrence of ‘leading to tray 2’ in each trial as the dependent variable. Finally, if the human (rather than the animal) took the lead, the subject could choose to follow or not. So we ran a final GLMM on a subset of the data (Total N. of trial for wolves and dogs =132) considering only trials in which the human lead the way from the first to the second tray and including whether the animal ‘followed the human to tray 2’ or not as the dependent variable. For all the above models both species and session were included as explanatory factors, animal Id was entered as random factor, and a binomial distribution was used.

All models were Generalized linear mixed model fit by maximum likelihood (Laplace Approximation). Models were run using the lme4 package in R (version 3.1.2, 2014) (Bates et al. 2013). All model assumptions were tested, and where overdispersion was found an observation level random effect was added. For all analyses to evaluate and compare different models, we used the Akaike Information Criterion (AICc), corrected for small sample sizes, and adopted a model averaging approach (using the package MuMin in R), allowing us to evaluate also the ‘relative importance’ of each variable across models (RVI) (^45-47^). Below tables with results from all models (combinations of variables tested) are presented.

**Tables Model comparisons**

**ST1: Model comparison for likelihood of success in the *spontaneous condition* (models are reported up to 3 AICc points from the best model).**

|  | df | logLik | AICc | delta | weight |
| --- | --- | --- | --- | --- | --- |
| Session & Prev. Success | 8 | -303.82 | 624.58 | 0.00 | 0.31 |
| Session, Prev. Success & Species | 9 | -303.64 | 626.47 | 1.89 | 0.12 |
| Session, Prev. Success & Training | 9 | -303.75 | 626.69 | 2.11 | 0.11 |
| Session | 7 | -306.02 | 626.77 | 2.19 | 0.10 |
| Session & Species | 8 | -304.99 | 626.93 | 2.34 | 0.10 |
| Prev. Success | 3 | -311.04 | 628.23 | 3.65 | 0.05 |

ST2: Model comparison for frequency of looking at the person in trials in which the *human* arrived first at the tray (models are reported up to 3 AICc points from the best model).

|  | df | logLik | AICc | delta | weight |
| --- | --- | --- | --- | --- | --- |
| Session, prev. success (& normalization time) | 4 | -322.31 | 652.70 | 0.00 | 0.44 |
| Session, prev. success, species (& normalization time) | 5 | -321.43 | 653.00 | 0.30 | 0.38 |
| Session, species (& normalization time) | 4 | -323.67 | 655.43 | 2.73 | 0.11 |

ST3: Model comparison for frequency of looking at the person in trials in which the *animal* arrived first at the tray (models are reported up to 3 AICc points from the best model).

|  | df | logLik | AICc | delta | weight |
| --- | --- | --- | --- | --- | --- |
| Species (& normalization time) | 3 | -315.13 | 636.33 | 0.00 | 0.24 |
| (normalization time) | 2 | -316.57 | 637.18 | 0.85 | 0.16 |
| Prev. success (& normalization time) | 3 | -315.66 | 637.39 | 1.06 | 0.14 |
| Session, species (& normalization time) | 4 | -314.73 | 637.59 | 1.26 | 0.13 |

**ST4: Model comparison for likelihood of the animal stealing the rope from the partner in trials in which the *human* arrived first at the tray (models are reported up to 3 AICc points from the best model).**

|  | df | logLik | AICc | delta | weight |
| --- | --- | --- | --- | --- | --- |
| Species & session | 4 | -196.70 | 401.48 | 0.00 | 0.60 |
| Species,Prev. success & session | 5 | -196.17 | 402.47 | 0.99 | 0.37 |
| Species | 3 | -201.21 | 408.47 | 6.99 | 0.02 |

ST:5 Model comparison for likelihood of success (solving both trays) in the dual tray condition.

|  | df | logLik | AICc | delta | weight |
| --- | --- | --- | --- | --- | --- |
| Session | 4 | -79.05 | 167.18 | 0.00 | 0.63 |
| Session & Species | 5 | -78.65 | 168.96 | 1.78 | 0.26 |
| (Null) | 3 | -82.33 | 171.29 | 4.11 | 0.08 |
| Species | 4 | -81.93 | 172.94 | 5.76 | 0.04 |

**ST6: Model comparison for likelihood of following the human partner to the first tray in the dual tray condition.**

|  | df | logLik | AICc | delta | weight |
| --- | --- | --- | --- | --- | --- |
| Session | 4 | -166.25 | 340.66 | 0.00 | 0.55 |
| Species& Session | 5 | -166.22 | 342.67 | 2.02 | 0.20 |
| (Null) | 3 | -168.38 | 342.86 | 2.21 | 0.18 |
| Species | 4 | -168.35 | 344.86 | 4.21 | 0.07 |

ST7: Model comparison for likelihood of the animal leading from the first to the second tray in the dual tray condition.

|  | df | logLik | AICc | delta | weight |
| --- | --- | --- | --- | --- | --- |
| Species & Session | 5 | -161.00 | 332.25 | 0.00 | 0.95 |
| Session | 4 | -165.47 | 339.10 | 6.85 | 0.03 |
| Species | 4 | -165.76 | 339.69 | 7.44 | 0.02 |
| (Null) | 3 | -170.23 | 346.56 | 14.31 | 0.00 |

**ST8: Model comparison for likelihood of the animal following the human from the first to the second tray in human-lead trials.**

|  | df | logLik | AICc | delta | weight |
| --- | --- | --- | --- | --- | --- |
| Species & Session | 5 | -78.81 | 168.10 | 0.00 | 0.59 |
| Session | 4 | -80.60 | 169.51 | 1.41 | 0.29 |
| Species | 4 | -81.84 | 171.99 | 3.90 | 0.08 |
| (Null) | 3 | -83.73 | 173.64 | 5.54 | 0.04 |

**Effect of experience with conspecifics of wolves in the dual tray condition**

Differently from dogs, a number of the tested wolves had already experienced the two apparatus condition with conspecifics ^8^, so it could be that the leading/following behavioural differences found between wolves and dogs were the result of such differences. A number of additional analyses were carried out to check for this possibility.

Firstly, we compared wolves and dogs only in the final session, and found that considering only this session wolves were still more likely to lead from tray 1 to tray 2 than dogs (wolves lead in 69% of trials compared to dogs leading in 47% of trials (χ2=3.9, p=0.048)). Furthermore, considering only trials in which the human led from tray 1 to tray 2, dogs were significantly more likely to follow than wolves (dogs followed in 90% and wolves in 39% of trials) (p=0.0031).

Second, we considered only wolves, which showed a range of experience in the two-tray condition with conspecifics (from animals that had no experience, because they had never been tested in this condition with a conspecific to animals successfully solving both apparatuses on 94% of trials). We ran a model evaluating whether leading/following behaviors in the two-tray condition with humans was affected by the animal’s previous success in the two-tray condition (i.e. percent of trials in which animals had successfully solved both trays with a conspecific).

There was no effect of previous success on leading from tray 1 to tray 2 (χ2=0.55, p=0.457), and wolves showed more leading behavior in later sessions (χ2=4.09, p=0.043). There was also no effect of previous success on following the human in trials in which the human lead (χ2=1.87, p=0.17), nor a session effect (χ2=0.05, p=0.82)

Taken together, these additional analyses suggest that the wolf-dog differences were not due to the potentially confounding factor of different levels of experience with the two-tray condition.

**Supplementary Material and Methods**

Subjects

Fifteen grey wolves (11 males, 4 females, age: 2 to 8 years) and 12 mixed-breed dogs (7 males, 5 females, age: 2 to 7 years) housed at the Wolf Science Centre (WSC) in Ernstbrunn, Austria, were tested. The wolves were born in reserves in Europe, America, Canada and Russia, whereas the dogs were born in animal shelters in Hungary with the exception of the 2014 dog generation, which was bred at the WSC (from two females with external mixed-breed males). Wolves and dogs were raised and are kept under the same conditions (see Range & Viranyi 2014 for details). At the age of 10 days, both wolves and dogs (except the 2014 generation) were separated from their mothers and hand-raised in groups. For the first 4 months, animals had continuous access to humans and adult pet dogs belonging to the hand-raisers. At 5 months of age, wolves and dogs were integrated into packs. The intense human contact in the first 5 months is necessary for socializing the wolves to be comfortable with humans. Initially this exact same procedure was used with the dogs. However, it was observed that some dogs may have found it challenging to then adjust to the reduced human contact and integration into conspecific packs. Therefore, for the most recent dog generation (2014 generation), the raising procedure was somewhat adapted to give the dogs more conspecific socialization. The puppies stayed in their natal packs overnight but spent several hours during the day with the human raisers and pet dogs. Due to their different predispositions towards conspecific and human interactions, this varied raising procedure likely results in comparable levels of human and conspecific socialization between wolves and dogs.

## Except three wolves, all animals tested in the current task had already participated in the same paradigm with conspecific partners ^8,43^ and had received different amounts of training as well as gained different experiences in the task. These aspects were controlled for in the statistical analyses (see below). The three naïve wolves received a minimum of training to pull a short rope with food attached through the fence of their home enclosure (independent of the table). Moreover, they were habituated to the movement of the tray without strings being present.

**ST9: Animals tested in the two conditions with their respective experiences. M = male, F = Female, No/yes indicates whether the animal was tested in the dual tray condition.**

| Name | **Species** | **Sex** | **Year Born** | **Dual tray** | **Training** | **% overall**  **previous success**  **with conspecific** |
| --- | --- | --- | --- | --- | --- | --- |
| amarok | Wolf | M | 2012 | No | Yes | 1,04 |
| kenai | Wolf | M | 2011 | Yes | Yes | 1,04 |
| aragorn | Wolf | M | 2008 | Yes | No | 68,05 |
| chitto | Wolf | M | 2012 | Yes | No | 62,03 |
| shima | Wolf | F | 2008 | No | No | 31,81 |
| tala | Wolf | F | 2012 | Yes | No | 63,36 |
| geronimo | Wolf | M | 2009 | No | Yes | 62,38 |
| yukon | Wolf | F | 2009 | Yes | Yes | 55,93 |
| kaspar | Wolf | M | 2008 | Yes | No | 65,04 |
| nanuk | Wolf | M | 2009 | Yes | Yes | 88,88 |
| una | Wolf | F | 2012 | No | Yes | 66,83 |
| wamblee | Wolf | M | 2012 | No | Yes | 9,44 |
| etu | Wolf | M | 2016 | No | No | 0 |
| maikan | Wolf | M | 2016 | No | No | 0 |
| tekoa | Wolf | M | 2016 | No | No | 0 |
| meru | Dog | M | 2010 | Yes | Yes | 30,55 |
| imara | Dog | F | 2014 | No | No | 4,16 |
| nuru | Dog | M | 2011 | No | Yes | 36,57 |
| pepeo | Dog | M | 2014 | Yes | No | 48,85 |
| panya | Dog | F | 2014 | Yes | No | 12,46 |
| maisha | Dog | M | 2009 | No | Yes | 0 |
| binti | Dog | F | 2010 | Yes | Yes | 0 |
| asali | Dog | M | 2010 | Yes | Yes | 0 |
| nia | Dog | F | 2011 | Yes | Yes | 1,39 |
| zuri | Dog | F | 2011 | No | Yes | 0 |
| sahibu | Dog | M | 2014 | Yes | No | 0 |
| gombo | Dog | M | 2014 | No | No | 0 |

## *Experimental set-up*

We conducted two experimental conditions: the *spontaneous* and *dual tray* conditions. All wolf-human and dog-human dyads received 6 sessions in the *spontaneous condition* consisting of 6 trials each to investigate how the animals reacted and performed when working with a human partner in a cooperative problem-solving task.

If the wolf/dog-human dyad successfully cooperated (criterion: at least 4/6 successful trials in at least two consecutive sessions), they immediately proceeded to the *dual tray condition* to test whether the animal would coordinate with the human partner not just in time, but also in space, by going first to one tray and then to the second. In this condition, each dyad received 3 sessions consisting of 6 trials each. All sessions were conducted on separate days with 2-3 days between sessions and lasted for 10- 15 minutes.

The tests were conducted in two different test enclosures at the WSC. The test enclosures were equipped with two shifting systems on opposite sides of the enclosure, each separated into 2-3 compartments, interconnected to each other and connected with the enclosure by multiple sliding doors. The sliding doors could be opened from outside the enclosure.

The tray consisted of a 1.5 x 0.75 m table with a wooden, flexible board mounted on top of it and 4 legs, adjustable in height. The table was located in one of the shifting compartments, separated from the test enclosure by a wire-mesh fence. The rewards were each located on two small wooden platforms, attached in the middle of the wooden board, 20 cm apart from each other. A 5.20 m long rope was looped through a pulling system to pull the board forwards and get access to the rewards. The ends of the rope were hanging down on each side with an approximate length of 1.20 m. The tray only moved if both ends of the rope were pulled simultaneously. If only one end of the rope was pulled, the rope would come loose and subjects would be unable to access the rewards.

During the test trials, the experimenter stood behind the compartment in which the test tray was located, obscured by a cover to avoid the subjects being influenced or distracted by her. Another helper was positioned behind the compartment, where the human and animal were located to release the subjects into the test enclosure during test trials (see supplementary figure 1a). During the *dual tray condition*, two trays were placed in different compartments of the shifting system, resulting in a distance of approximately 10 m between them. Additionally, another helper was standing behind the second tray also concealed by a cover (see supplementary figure 1b).


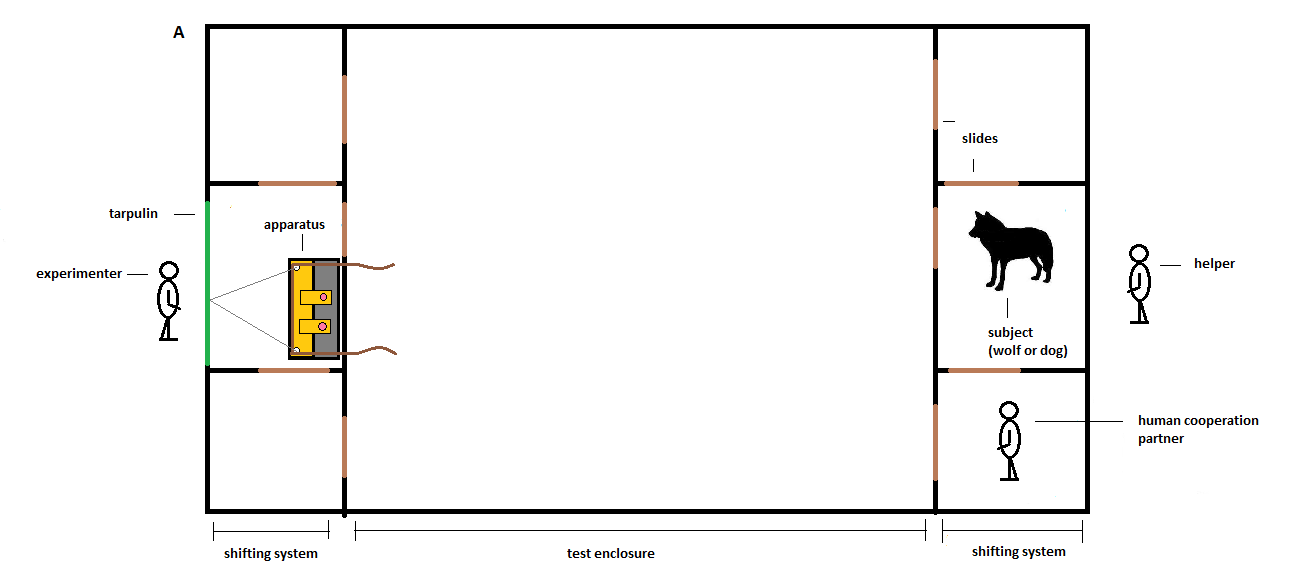


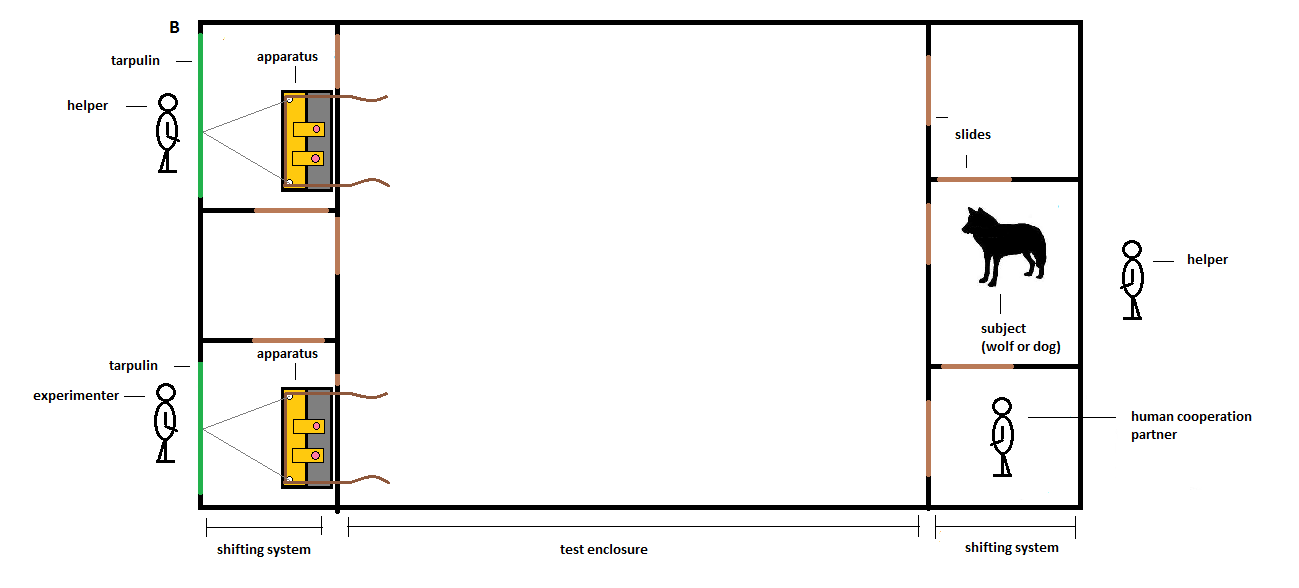


Figure S1: a: Illustration of the experimental setting for the *spontaneous condition*. b: Illustration of the experimental setting for the *dual tray condition (drawing by Corinna Kratz).*

*Procedure spontaneous condition*

At the start of each test trial, the animal subject (either wolf or dog) and the human cooperation partner were positioned in the middle compartment of the shifting system facing the test tray at a distance of 40 m. The animal and human had one minute to greet each other in the middle compartment, before the human was shifted into an adjacent compartment so that the subjects could be released individually. This procedure was chosen to avoid creating a delay condition unintentionally, since the human needed approx. 12 seconds to cover the distance to the tray, while the animal needed only about 4 seconds. Accordingly, in 50% of the trials, we released the animal when the human partner had reached the middle of the enclosure so that the animal reached the tray approximately 2-3 seconds before the human cooperation partner. In these trials, the animal could choose the side it wanted to pull the rope on, but only needed to wait shortly for the human partner. In the other 50% of the trials, the animal was released when the human cooperation partner was only 3 meters away from the tray, so that the human cooperation partner arrived approx. 3 seconds (counted in the head) before the animal at the tray and could choose the side to pull the rope on. For animals that had a side preference based on the trials with conspecific partners, the human always chose the preferred side of the animal, when she was first at the tray so that overall the subject had to pull 3 times on each side. If animals did not have a side preference (N = 10 animals), the human chose the side the animal had pulled the rope on in the previous trial. This procedure assured that at least in half of the trials, the animals had to adjust to the human cooperation partner and not vice versa.

At the beginning of each test trial, the experimenter prepared the tray and placed the rope in the test enclosure, guaranteeing that both ends were of the same length. Subsequently, the experimenter turned towards the subjects (wolf or dog and the human cooperation partner), called their names and at the same time showed them two pieces of sausage (each in one hand), to get the subjects’ attention. Afterwards, the experimenter put the two pieces of sausage on the respective platforms and left the test enclosure. As soon as the experimenter was positioned behind the test tray and gave the ‘O.K.’ signal to the helper, the subjects were released according to the release schedule.

The human cooperation partner followed the subsequent protocol:

1. The human cooperation partner did not talk, look or gesture to the animal in any way during a trial.
2. From the moment that the door opened the human cooperation partner walked at a normal pace directly towards the middle of the tray.
3. If the human cooperation partner approached the tray first, she chose the rope to pull according to a pre-set sequence that balanced the sides so that each partner pulled the rope three times on each side. Following this, the human cooperation partner picked up the rope, held the rope for three seconds and then, independently of the animal’s behaviour, started to pull the rope, slowly and continuously.
4. If the animal did not approach the tray in time or did not take the other rope, the human cooperation partner pulled the rope through and the trial ended.
5. If the animal approached the tray before the 3 seconds passed, took the other rope and started to pull, the human cooperation partner pulled the rope as well.
6. In cases in which the animal decided to pull the same rope the human partner had already chosen to pull, the human cooperation partner continued holding it for a few seconds and then released it and stepped aside in order to avoid any competition and potential conflicts with the subjects. If this occurred, the trial ended.
7. If the animal approached the tray first, the human cooperation partner chose the rope not chosen by the animal. If the animal did not pull the rope at that time, the human cooperation partner picked up her rope and held it, without pulling it for 3 seconds and then pulled it through, slowly and continuously. If the animal had already started pulling the rope by the time the human cooperation partner approached her side of the tray, she picked up the other rope (if it was still available) and pulled together with the animal.
8. If the subjects pulled the rope together, but the animal stopped, the human cooperation partner kept pulling the rope out.
9. If the partners successfully pulled the tray forward, the human tried to secure her reward as fast as possible. However, if the animal tried to obtain also the partner’s reward, the human cooperation partner stepped aside to avoid any competition and potential conflicts.
10. After a trial ended, the subjects were called back to the shifting system by the helper and the next trial started.

If an animal did not pull on a rope in any trial during a session (N=4), a motivation session was included afterwards. During these motivation trials, a piece of meat, directly attached to the rope, was placed on the tray, alternating the position between the two food slots. Animals had to successfully retrieve the meat in three consecutive trials by pulling the rope out with no prompting and/or encouragement.

## *Procedure dual tray condition*

At the beginning of each test trial, the experimenter prepared the trays similarly to the *spontaneous condition* (see above). The order in which the experimenter prepared the trays was randomized. In this condition, the human was always released first and the animal was released once the human had reached the middle of the enclosure. The human partner approached the trays from the middle, only starting to directly approach the *a priori* designated tray (pseudo-randomized), when reaching the second half of the enclosure. In the first two trials, the human cooperation partner did not approach the same tray and in subsequent trials she did not choose the same tray more than twice in a row. Hence, in some of the trials the animal was forced to switch from one to the other tray to successfully solve the task with the human partner.

The general procedure was identical to the *spontaneous condition,* however, the following points were changed in the protocol for the human partner.

1. If the animal approached the first assigned tray first, the human partner took the rope not chosen by the animal.
2. If the human cooperation partner approached the first tray before the animal, she chose the side of the rope ***not*** preferred by the animal based on side preferences observed in the previous condition. Since in this condition we were primarily interested, whether the animals would coordinate their behaviour with the human partner and approach the same tray, we opted not to force them to also use their non-preferred side as in the spontaneous condition.
3. If the human cooperation partner and the animal solved the first tray successfully or if the rope was pulled out or stolen by one of the cooperation partners, the human cooperation partner either 1) followed the animal, if it moved towards the second tray or 2) waited for 5 seconds (counted in the head) before approaching the second tray.
4. The human cooperation partner again walked at a normal pace and did not communicate (talking, looking, gesturing) towards the animal in any possible way.

## *Behavioural analysis*

All experiments were videotaped using a camcorder for behaviour analysis in Solomon Coder (Péter 2011; [https://solomoncoder.com](https://solomoncoder.com/), version beta17.03.22). Definitions of coded behaviours can be found in Table ST10.

ST10: Definitions of coded behaviours

| **Behaviour** | **Measure** | **Definition** |
| --- | --- | --- |
| **Spontaneous Condition** |  |  |
| Success | Occurrence (yes/no) | The animal and human partner simultaneously pull on the rope, moving the tray forward so that the food is within reach. |
| Proximity to tray | Duration | Time spent by the animal less than 1 body length from the apparatus. |
| Gaze at human | Frequency | N of times the animal changes its head orientation so as to look at the human partner. |
| Steal rope from the human | Occurrence  (yes/no) | The animal takes the rope end held by the human cooperation partner in its mouth and does not let go within 2 seconds; the human cooperation partner therefore drops the rope and retreats. |
| **Dual Apparatus Condition** |  |  |
| Success | Occurrence (yes/no) | In a trial the animal and human partner succeed in pulling both trays forward so that the food is in reach (as above, by simultaneously pulling on the rope) |
| Following human to tray 1 | Occurrence (yes/no) | The animal directly approaches the same tray the human cooperation partner has reached (without first going to the other tray). |
| Leading to tray 2 | Occurrence (yes/no) | After completion of tray 1 (regardless of success) the animal moves away from tray 1 and towards tray 2 ahead of the human partner. |
| Following human to tray 2 | Occurrence (yes/no) | After completion of tray 1 (regardless of success) and after 5 seconds have elapsed the human cooperation partner goes to tray 2 and the animal (which is either still at tray 1 or has walked in any other direction than tray 2) directly follows her staying within one body length. |

Interobserver reliability was high for all behavioural variables analysed (Interclass correlation coefficient: success= 0.92; gazing at the human= 0.91, proximity to tray= 0.89; stealing the rope= 1).

ST11: mean and SD for analysed behaviours in the Spontaneous condition for wolves and dogs

| **Behaviour** | **Species** | **mean** | **SD** |
| --- | --- | --- | --- |
| Success (N. of trials per session) | wolf | 3.67 | 1.98 |
|  | dog | 2.92 | 2.22 |
| Gaze human (frequency per trial) | wolf | 0.29 | 0.75 |
|  | dog | 0.74 | 1.32 |
| Steal rope (N of trials per session) | wolf | | 0.46 | | --- | | |  | | --- | |  | |  | | | 0.78 |
|  | dog | \| 0.05 \| \| --- \| \| \|  \| \| --- \| \|  \| \|  \| \| | 0.21 |

ST12 : mean and SD for coded behaviours in the Two-tray condition for wolves and dogs

| **Behaviour** | **Species** | **mean** | **SD** |
| --- | --- | --- | --- |
| Success (N. of trials per session in which both trays were successfully solved) | wolf | 4.57 | 1.47 |
|  | dog | 4 | 1.67 |
| Follow human to tray 1 (N. of trials per session) | wolf | | 3.71 | | --- | |  | | 1.31 |
|  | dog | 3.52 | 1.23 |
| Leading to tray 2 (N. of trials per session) | wolf | | 3.57 | | --- | |  | | 1.57 |
|  | dog | \| 2.09 \| \| --- \| \|  \| | \| 1.18 \| \| --- \| |
| Following human to tray 2 (when human leads) (N. of trials per session) | wolf | | 1 | | --- | |  | | 1.14 |
|  | dog | \| 2.7 \| \| --- \| \|  \| | 1.17 |

Movie S1: Dog cooperating with a human partner in the one tray condition

Movie S2: Wolf Cooperating with a human partner in the one tray condition
